# Computational identification and experimental validation of PPRE motifs in NHE1 and MnSOD genes of Human

**DOI:** 10.1186/1471-2164-10-S3-S5

**Published:** 2009-12-03

**Authors:** Gireedhar Venkatachalam, Alan Prem Kumar, Loo Ser Yue, Shazib Pervaiz, Marie Veronique Clement, Meena Kishore Sakharkar

**Affiliations:** 1 Department of Biochemistry, Yong Loo Lin School of Medicine, National University of Singapore, Singapore; 2 National University Medical Institutes, Yong Loo Lin School of Medicine, National University of Singapore, Singapore; 3 Department of Physiology, Yong Loo Lin School of Medicine, National University of Singapore, Singapore; 4 NUS Graduate School for Integrative Sciences and Engineering, National University of Singapore, Singapore; 5 Duke-NUS Graduate Medical School, Singapore, Singapore; 6 Singapore-MIT Alliance, Singapore; 7 Biomedical Engineering Research Centre, Nanyang Technological University, Singapore; 8 Advanced Design and Modeling Lab, Nanyang Technological University, Singapore

## Abstract

**Background:**

Activation of PPARs has been reported to inhibit the proliferation of malignant cells from different lineages. They are involved in transcription regulation of genes upon activation by a ligand. The binding of PPARs to the promoter sequence either represses or activates the gene. Hence, PPARs represent promising targets for cancer treatment because of their anti-proliferative and pro-apoptotic activities. Here we computationally identified PPAR binding regions in NHE1 and MnSOD. We further validated the predictions in vitro.

**Results:**

Our results computationally predicted the presence of 2 PPRE motifs in NHE1 and 3 PPRE motifs in MnSOD. We experimentally confirmed the true motifs and their regulation by PPAR.

**Conclusion:**

Our results suggest that both NHE1 and MnSOD have PPRE binding motif in their upstream/promoter region and hence are regulated by PPAR upon ligand binding.

## Introduction

PPARs (Peroxisome proliferators activated Receptor) belong to the nuclear receptor super family and are ligand activated transcription factors, regulating a wide variety of genes [1]. Phylogenetic studies have shown that PPARs form a subfamily of the nuclear receptor superfamily, along with the receptors for thyroid hormone, retinoic acid (RA), vitamin D, ecdysone, and the orphan receptors Rev-ErbAa (5ear1;NR1D1) and E75 (NR1D3, from Drosophila), the two latter being the closest relatives of the PPARs [1]. PPARs play important role in lipid metabolism, cell differentiation and development, inflammation, cancer and demyelination [2].

Three isoforms (α, β and γ) for PPAR have been identified so far in xenopus, mouse, human, rats and hamsters [3]. The isoforms exhibit a tissue specific expression pattern. The first isoform PPAR α is expressed in tissues that play a role in fatty acid catabolism such as liver, kidney, heart, and intestine [4]. This isoform is the first described receptor that is activated by peroxisome proliferators and hence the name [5]. The main function of this isoform is to regulate the genes encoding lipid metabolizing enzymes and proteins participating in lipid metabolism [4]. The second isoform is PPAR β. This receptor is also called as FAAR (fatty acid activated receptor)[1]. This isoform is expressed mainly in the brain, skeletal muscle, skin, adipose tissue, gut, placenta and has a very broad expression pattern. Based on the cell proliferation and differentiation, the expression profile of this isoform varies. Both isoforms PPAR α and β are involved in lipid utilization activities such as fatty acid oxidation, energy uncoupling reactions and response to fasting [1]. PPAR γ has three alternatively spliced isoforms. PPAR γ1 is virtually expressed in almost all tissues [6]. PPAR γ2 is primarily expressed in adipose tissue while PPAR γ3 is expressed in white adipose tissue, large intestine and macrophages. PPAR γ regulates the lipid storage and insulin sensitivity reactions such as lipogenesis, adipocyte differentiation, adipocyte survival and adipokine secretion [1]. Activation of the peroxisome proliferator-activated receptor-γ (PPARγ) has been identified as an approach to induce differentiation and inhibit proliferation in a variety of cancer cells. Breast tissue, in particular, was found to express PPARγ in amounts greater than those found in normal breast epithelium [7]. Moreover, activation of PPARγ through cells' exposure to synthetic PPARγ ligands such as proglitazone or rosiglitazone is shown to exert antitumor activity through cell growth inhibition and cellular differentiation. Activation of PPARs has been reported to inhibit the proliferation of malignant cells from different lineages such as liposarcoma, breast adenocarcinoma, prostate carcinoma, colorectal carcinoma, non-small cell lung carcinoma, pancreatic carcinoma bladder carcinoma, and gastric carcinoma both *in vitro *and *in vivo*.

**Figure 1:**
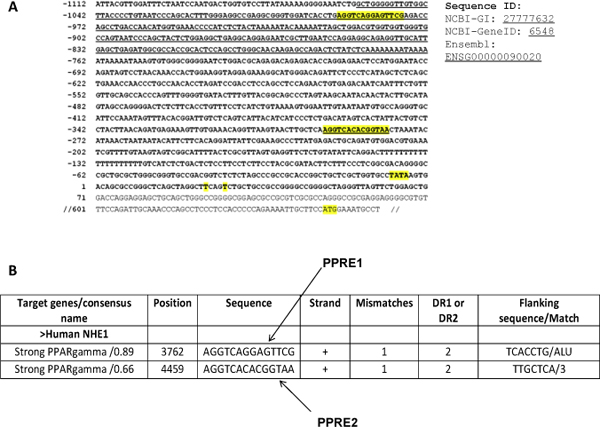
**Identification of putative PPREs in promoter of NHE1 and MnSOD**. (A) Human NHE1 promoter sequence from NCBI and Ensembl (Sequence IDs indicated). Alu element underlined, with putative PPREs italicized and bold. Transcriptional start site (T) and ATG translational start codon shown in figure. (B) We predicted two PPREs with PPARγ binding efficiencies indicated.

**Figure 2:**
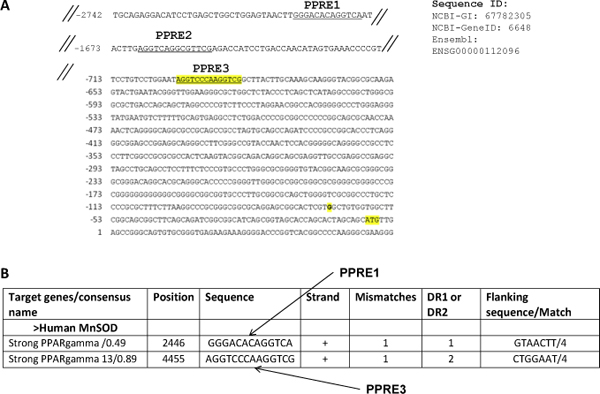
**Identification of putative PPREs in promoter of NHE1 and MnSOD**. (A) Human MnSOD promoter sequence from NCBI and Ensembl (Sequence IDs indicated). Alu element underlined, with putative PPREs italicized and bold. Transcriptional start site (G) and ATG translational start codon shown in figure. (B) We predicted PPRE1 and PPRE3 with PPARγ binding efficiencies indicated.

Various studies have also shown that PPARs are involved in transcription regulation of genes upon activation by a ligand. The binding of PPARs to the promoter sequence either represses [8] or activates [9] the gene. Hence, PPARs represent promising targets for cancer treatment because of their anti-proliferative and pro-apoptotic activities. The PPAR α, β, and γ isoforms share a highly conserved DNA binding domain that recognizes specific DNA sequences known as Peroxisome Proliferator Response Elements (PPREs) (11). PPAR DNA-binding activity is modulated by Retinoic-X-Receptor (RXR). Upon ligand binding, PPAR translocates from cytoplasm to nucleus and forms heterodimer with RXR. PPAR/RXR complex then binds to PPRE composed of a Direct Repeat (DR) preferably spaced by one nucleotide (DR1) with a consensus sequence of AGGTCA-A-AGGTCA. It must be noted that there are DR2 motifs which are also recognized by PPAR that are preferably spaced by two nucleotide (DR2). Experimental evidences link upregulation of the pH regulator Na^+^/H^+ ^exchanger 1 (NHE1) to the development and progression of carcinogenesis [10-15] and its down regulation to inhibition of cells' growth and enhanced apoptotic sensitivity [16,17]. It is also suggested that cancer cells are generally under reactive oxygen species (ROS) stress [18]. As mitochondrial respiration is the main source of O_2_^•- ^generation in the cells, MnSOD (manganese superoxide dismutase) is of prime importance in maintaining cellular ROS balance and mitochondrial integrity in cells. This is specifically true for tumor cells which are constantly under ROS stress due to its increased metabolic processes [20]. Considering the abundance of PPARγ receptor and elevated NHE1 expression in a variety of cancer cells including breast cancer and their absolute dependence on MnSOD it is of interest to explore PPARγ agonists for selectively tailoring the expression of NHE1 and MnSOD. Therefore, we manually collected experimentally proven PPRE binding motifs from literature and arrived at a collection of 250 motifs. Search of human NHE1 and MnSOD promoter sequence against this collection predicted two putative DR2 PPRE motifs in NHE1 (Figure 1) and one DR1 PPRE and two DR2 PPRE motifs in MnSOD (Figure 2). We further investigated if PPARγ binds to the promoter of NHE 1 and MnSOD genes at these PPRE motifs. Since these genes have not been identified as PPARγ target genes in breast cancer cells, the results for the first time confirm regulation of NHE1 and MnSOD by PPARγ.

## Methodology

### Collection of PPRE motifs from literature

This collection contains 250 reported PPRE motifs from literature. Keywords "PPRE", "PPAR consensus sequences" "PPAR binding sequences" were used to search for literatures in pubmed and Google Scholar. The sequences reported only with experimental validation were added to this collection. For each PPRE data on its reported consensus, isoform specificity, in vivo and in vitro binding efficiencies and Pubmed IDs were collected (additional file 1).

### Promoter sequence extraction and motif identification

The promoter sequences (5 kb) upstream of the transcription start site from transcripts of NHE1 and MnSOD were downloaded from NCBI http://www.ncbi.nlm.nih.gov using keywords NHE1 and MnSOD. The motif collection was searched/matched (string comparison) by a cgi-script against the extracted sequence of NHE1 and MnSOD, to check if there is presence of any of the motifs (string) in the upstream regions of the above gene sequences. Search of human NHE1 and MnSOD promoter sequences against this collection predicted/identified two putative DR2 PPRE motifs in NHE1 (Figure 1) and one DR1 PPRE and two DR2 PPRE motifs in MnSOD (Figure 2).

The general DR1 and DR2 consensus are AGGTCA N AGGTCA (6-N-6) and AGGTCA NN AGGTCA (6-NN-6), respectively. Both the above patterns were matched against the collection, first by looking for the surrounding hexamers and then looking for the spacers i.e. one nucleotide or 2 nucleotides separation. Thus, the motifs for DR1 and DR2 are identified based on the number of 'N's that separate the two hexamer consensus pattern.

These predicted motifs were experimentally validated as explained in the subsequent sections.

### Validation of identified patterns in NHE1 and MnSOD

#### Reagents and antibodies

Roswell Park Memorial Institute (RPMI) 1640 medium, phosphate buffered saline (PBS), fetal bovine serum (FBS), L-glutamine and trypsin was purchased from Hyclone UT, USA. Pepstatin A, phenylmethanesulfonyl fluoride (PMSF), leupeptin, bovine serum albumin (BSA), mouse anti-β-actin monoclonal antibody were supplied by Sigma-Aldrich LO, USA. Aprotinin was purchased from Applichem Darmstadt, Germany. Mouse anti-HuNHE1 monoclonal antibody was purchased from Chemicon International MA, USA. Rabbit anti-HuMnSOD monoclonal antibody was purchased from Upstate, NY, USA. Stabilized goat anti-mouse horseradish peroxidase (HRP) was obtained from Pierce IL, USA. The PPARγ agonist, 15 d-PGJ_2 _was purchased from Alexis Biochemical CA, USA. 5'-biotinylated oligonucleotides for NoShift assay were synthesized by Proligo, Singapore.

#### Cell lines and culture conditions

Human MCF-7, MDA-MB-231, and MDA-MB-468 breast carcinoma cells were obtained from the American Type Culture Collection (ATCC MD, USA). MCF-7, MDA-MB-231, and MDA-MB-468 cells were propagated and maintained in RPMI medium containing 10% fetal bovine serum (FBS), 2 mM L-glutamine, and 1 mM gentamicin sulfate at 37°C and 5% CO_2_. Cultures were replenished with fresh medium every 2 to 3 days and passage 1:3 when they reached 80% confluence.

#### Western blot analysis

Whole cell lysates were prepared with RIPA lysis buffer containing 10 mM Tris-HCL pH7.4, 30 mM NaCl, 1 mM EDTA, 1% Nonidet P-40, supplemented with 1 mM Na_3_VO_4_, 1 μg/ml leupeptin, 1 μg/ml pepstatin A, 1 μg/ml aprotinin and 1 mM PMSF before use. Protein concentration was determined for each sample and equal amounts of protein were warmed at 37°C in the water bath for NHE1 protein, boiled for 5 min for MnSOD, with 1 × SDS sample buffer and resolved by 8% (NHE1) or 12% (MnSOD) SDS-PAGE. Thereafter, proteins were transferred onto nitrocellulose membrane, blocked for 1 h at RT with 5% non-fat milk, and incubated overnight at 4°C with the primary antibody. After probing with secondary antibody for 1 h at 25°C, protein bands were detected by using the Supersignal West Pico Chemiluminescence (Pierce IL, USA). β-actin antibody was used as a loading control.

#### Reporter plasmid constructs

Chloramphenicol acetyltransferase (CAT) pUCSS-CAT reporter plasmid constructs: -1374/+16, -850/+16, and empty vector pUCSS-CAT were kindly provided by Dr. Alexey Kolyada, Dept of Medicine, Tufts University School of Medicine, Boston, USA (Kolyada et al., 1994). Full length and 5' deletion constructs of the human MnSOD promoter tagged to luciferase reporter gene were a gift from Dr. Daret K. St Clair, Graduate Center for Toxicology, University of Kentucky, USA (Xu et al., 2002).

#### Chloramphenicol acetyltransferase (CAT) ELISA

The levels of CAT protein were quantified using a CAT antigen capture enzyme-linked immunosorbent assay (ELISA) (Roche Molecular Biochemicals Mannheim Germany). All CAT quantitations were normalized to the protein concentration of the cell extract, as determined using the Coomasie Plus Protein Assay Reagent Kit (Pierce IL, USA).

#### Luciferase reporter assays

3 × PPRE promoter activity was assessed with a dual-luciferase assay kit (Promega WI, USA). Briefly, feeding medium was removed from the wells, washed once with 1× PBS, and lysed with ice-cold 100 μl of reporter lysis buffer. Ten microlitres of cell lysate was then added to 50 μl of luciferase substrate solution, following which 50 μl of stop & glow buffer was added for Renilla reading. Bioluminescence generated was measured using a Sirius luminometer (Berthold Technologies, Deutschland, Germany). The luminescence readings obtained were normalized to the protein content of the corresponding cell lysate. Activity was calculated by dividing luminescence readings expressed in relative light units (RLU)/renilla/μg total protein in 10 μl of cell extract used in the assay.

#### NoShift transcription factor assay

Nuclear and cytosol fractions of MDA-MB-231 cells treated with 15 d-PGJ_2 _were prepared using the NE-PER Nuclear and cytoplasmic Extraction kit (Pierce IL, USA). The binding affinities of PPARγ protein in nuclear extracts to three target PPREs on MnSOD promoter were determined with the NoShift transcriptional factor assay kit (Novagen Inc. NJ, USA). The oligonucleotide sequences (listed below) used for the three putative PPREs on MnSOD were first annealed in a PCR machine with 1× annealing buffer (1 mM TrisHCl pH 8, 50 mM NaCl, 10 mM MgCl_2_) and the annealed oligonucleotides then diluted to a final concentration of 100 ng/μl.

HuMnSODPPRE-1 Sense 5'-Biotin-GTAACTTGGGACACAGGTCAATCGACTG-3'

HuMnSODPPRE-1 Antisense 5'-Biotin-CAGTCGATTGACCTGTGTCCCAAGTTAC-3'

HuMnSODPPRE-2 Sense 5'-Biotin-CACTTGAGGTCAGGCGTTCGAGACCA-3'

HuMnSODPPRE-2 Antisense 5'-Biotin-TGGTCTCGAACGCCTGACCTCAAGTG-3'

HuMnSODPPRE-3 Sense 5'-Biotin-TGGAATAGGTCCCAAGGTCGGCTTAC-3'

HuMnSODPPRE-3 Antisense 5'-Biotin-GTAAGCCGACCTTGGGACCTATTCCA-3'

For measurement of PPARγ binding, each reaction mixture contained 5 μl of 4× NoShift Bind buffer, 1 μl of poly(dI-dC)·poly(dI-dC) (0.01 U/μl in 100 mM KCl, 20 mM HEPES, pH 8.0), 1 μl of salmon sperm DNA (500 ng/μl in nuclease-free water), 1 μl of biotinylated 10-pmol/μl target DNA duplex, and 20 μg of nuclear lysate in a total reaction volume of 20 μl. After 30 min incubation on ice, 80 μl of 1× NoShift Bind buffer was added to each reaction mixture, and the resulting 100 μl was dispensed into one well of a freshly washed streptavidin plate and incubated for 1 h at 37°C. After this incubation period, plates were washed with 1× NoShift Bind buffer, the binding of PPARγ was detected by incubation for 1 h at 37°C with 100 μl of anti-PPARγ diluted 1:250 in NoShift antibody dilution buffer. After repeated washings, horseradish peroxidase (HRP)-conjugated anti-rabbit immunoglobulin G (IgG) was added (1:1000 dilution in NoShift antibody dilution buffer). After 30 min of incubation at 37°C, wells were washed thoroughly. Finally, 100 μl of room temperature TMB (tetramethylbenzidine) substrate was added and the wells were incubated for 10-30 min at room temperature in the dark until a blue color developed. The reaction was stopped by adding 100 μl of 1 M HCl to each well, and *A*_450 _was measured using Spectrofluoro Plus spectroflurometer (TECAN, Grodig, Austria).

#### DNA transfections

Cells were transfected using CalPhost Mammalian transfection kit (BD Bioscience, CA, USA) for 15 h. Cells were then washed twice with PBS and culture media added for another 24 h. Co-transfection with the *Renilla *plasmid (Clontech, CA, USA) was used to assess transfection efficiency in dual-luciferase reporter assay (Promega, WI, USA).

#### RNA isolation and mRNA determination by real-time PCR

Total RNA was isolated from cells by TRIZOL reagent (Invitrogen, CA, USA) as described by manufacturer's instructions with a DNAse treatment step incorporated into the protocol. Each RT reaction contains 2.5 μg of total RNA, 1× RT buffer, 5 mM MgCl_2_, 425 μM each of dNTPs, 2 μM random hexamers, 0.35 U/μl RNase inhibitor, 1.1 U/μl MultiScribe™ reverse transcriptase and made up to 10 μl with sterile water. RT reaction was carried out at 37°C for 1 h. 5 μl of the 10 μl cDNA reaction volume was used in realtime quantitative PCR using ABI PRISM 7500 (Applied Biosystems, CA, USA). Normalization was to either glyceraldehyde 3-phosphate dehydrogenase (GAPDH) or 18S RNA. Fluorescence was measured with the Sequence Detection Systems 2.0 software. PCR was performed in multiplex (both target and endogenous control co-amplified in the same reaction with distinct fluorescent dyes. Primers and probe for human glyceraldehyde-3-phosphate dehydrogenase (GAPDH), 18S RNA, human NHE1 and human MnSOD were purchased as kits from Applied Biosystems (Assays on Demand).

### Statistical analysis

Statistical analysis was performed using paired Student's t-test. A p-value of less than 0.05 was considered significant.

## Results

### PPRE motif collection

It is known that the general DR1 and DR2 consensus are AGGTCA N AGGTCA (6-N-6) and AGGTCA NN AGGTCA (6-NN-6), respectively. The determining factor for PPAR binding to the PPRE are the surrounding hexamers 1 and 2 (AGGTCA). The DR1 and DR2 motifs share the same hexamers and PPARs have also been reported to bind hexamers with DR2 spacers. Thus, for PPARs optimal spacer nucleotide is either 1 or 2. Out of the 413 PPRE DR1 motifs collected from literature the binding strengths were reported for 236 motifs (Additional file 1). These 236 motifs were used to match for both DR1 and DR2 sequences. Based on sequence match, we identified two putative PPREs in NHE1 and three putative PPREs in MnSOD promoter sequences.

### Identification of Peroxisome Proliferator Response Elements (PPREs) within the human NHE1 and MnSOD promoters

We identified two putative PPREs in the human NHE1 promoter, (Figure 1). The first PPRE site (PPRE1) is within a primate-specific Alu element (Sq) [21], within which there is a cluster of four hexamers half-sites recognized by various nuclear receptors [22-25], termed as Alu receptor response element (AluRRE). Kumar et al. have previously shown in the context of the human myeloperoxidase gene, a DR2 element is also recognized by PPARγ [24]. DR2 element, in both PPRE1 and PPRE2 are seen here within the human NHE1 promoter. We identified three putative PPREs in the human MnSOD promoter region (Figure 2C). In the human MnSOD promoter, PPRE1 is a DR1 element, while PPRE2 and PPRE3 are of the DR2 elements. Here, PPRE2 is within a primate-specific Alu element (Sx) [21].

### PPARγ activation by 15 d-PGJ_2 _represses NHE1 mRNA and protein levels

**Figure 3:**
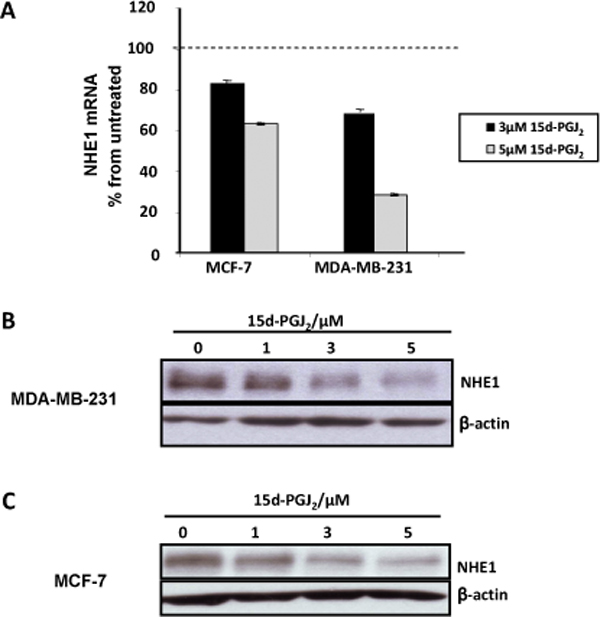
**Activation of PPARγ induces NHE1 gene repression**. (A) MCF-7 and MDA-MB-231 cells were exposed to 3 μM and 5 μM 15 d-PGJ_2 _for 24 h before NHE1 mRNA and protein levels were assessed as described in M&M. Relative NHE1 mRNA expression is expressed as percent of untreated control. Data shown is the mean of three experiments done in duplicate +/- SD. NHE1 protein expression in (B) MCF-7 and (C) MDA-MB-231 was analyzed by Western blot following 15 d-PGJ_2 _at doses indicated for 24 h. Equal loading ascertained using detection of β-actin.

To assess the effect of PPARγ ligand (**15 d-PGJ**_2_) on NHE1 gene expression, MCF-7 and MDA-MB-231 cells were treated with 3 μM and 5 μM 15 d-PGJ_2 _for 24 h. 15 d-PGJ_2 _(15-deoxy-Δ^12,14^-PGJ_2_) is a metabolite of the eicosanoid prostaglandin J_2_, and is described as the most potent natural ligand for PPARγ with reported K_d_s varying from 325 nM to 2.5 μM. As shown by real time quantification of mRNA level, exposure of both cell lines to increasing concentration of 15 d-PGJ_2_, led to a significant decrease in NHE1 mRNA levels (Figure 3A) dose-dependent decrease in NHE1 protein expression (Figure 3B and 3C).

### PPARγ activation by 15 d-PGJ_2 _represses MnSOD mRNA and protein levels

**Figure 4:**
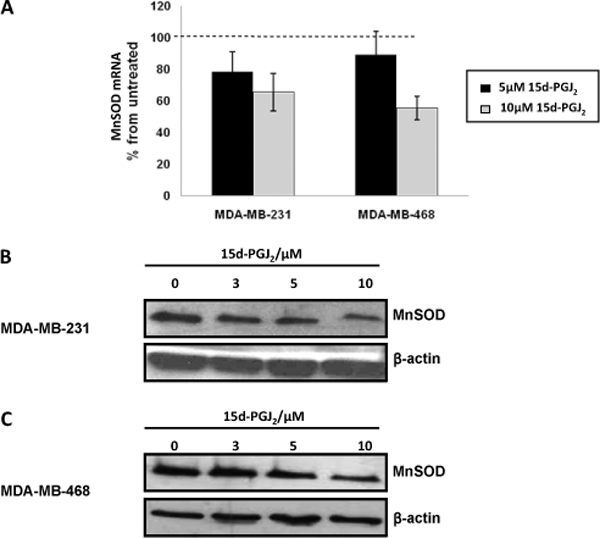
**Activation of PPARγ represses MnSOD gene repression**. (A) MDA-MB-231 and MDA-MB-468 cells were exposed to 5 μM and 10 μM 15 d-PGJ_2 _for 24 h before MnSOD mRNA and protein levels were assessed as described in M&M. Relative MnSOD mRNA expression is expressed as percent of untreated control. Data shown is the mean of three experiments done in duplicate +/- SD. MnSOD protein expression in (B) MDA-MB-231 and (C) MDA-MB-468 was analyzed by Western blot following 15 d-PGJ_2 _at doses indicated for 24 h. Equal loading ascertained using detection of β-actin

We investigated the regulation of human MnSOD mRNA and protein levels in two breast cancer cell lines treated with 15 d-PGJ_2_. Realtime-PCR analysis shows that relative MnSOD mRNA expressions in both MDA-MB-231 and MDA-MB-468 cells were significantly reduced when exposed varying 15 d-PGJ_2 _doses (Figure 4A). Western blot analysis corroborated this decrease in MnSOD mRNA levels with an observed dose-dependent decrease in MnSOD protein levels in MDA-MB-231 and MDA-MB-468 cells (Figure 4B and 4C).

### Experimental evidence for PPRESearch-derived prediction for PPARγ binding site in NHE1 promoter

**Figure 5:**
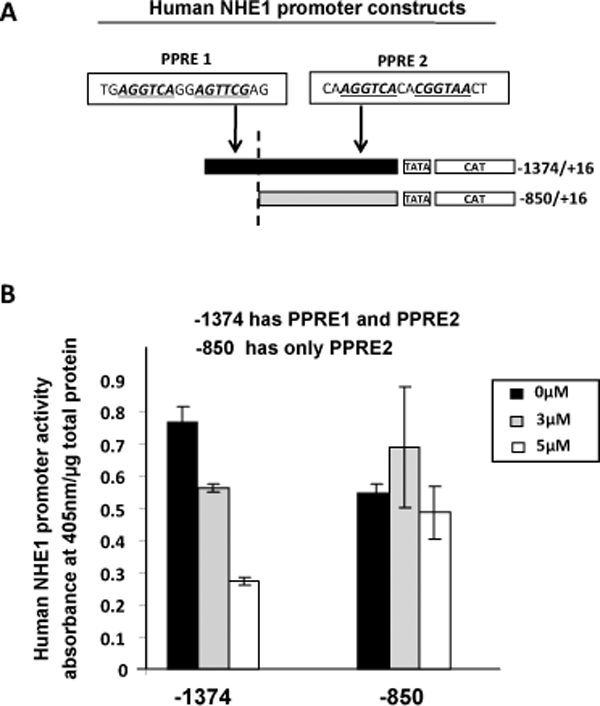
**PPARγ binds to PPRE1 in human NHE1 promoter**. (A) Schematic representations of full length human NHE1 promoter construct (-1374/+16) and a 5'-deletion construct (-850/+16) with location of PPRE1 and PPRE2 indicated. (B) MCF-7 cells transiently transfected with full length human NHE1 promoter construct (-1374/+16) and a 5'-deletion derivative of the full length lacking the PPRE site (-850/+16). Cells were treated with 3 μM and 5 μM 15 d-PGJ_2 _for 16 h. CAT-ELISA activity measured following manufacturer's instructions. NHE1 promoter activities were calculated as CAT activity (A_405 nm_)/μg total protein and expressed as absorbance/μg total protein. Data represents the average +/- SD of two experiments done in duplicate.

To determine which of the two PPREs within the human NHE1 is the true PPARγ binding site, we transfected MCF-7 breast cancer cells with a full length NHE1 promoter construct (containing both PPRE1 and PPRE2) compared to a parallel set up transfected with a 5' deletion of the full length (only contains PPRE2) (Figure 5A). Activation of PPARγ by its natural ligand, 15 d-PGJ_2 _decreased NHE1 promoter activity only when cells were transfected with the full length NHE1 promoter construct but not when PPRE1 is absent (Figure 5B). Our results therefore demonstrate that NHE1 is a PPARγ target gene. We also narrowed the bonafide PPARγ binding site to PPRE1, thereby providing experimental evidence for predicted PPRE motifs in the upstream region of NHE1.

### Experimental evidence for PPRESearch-derived prediction for PPARγ binding site in human MnSOD promoter

**Figure 6:**
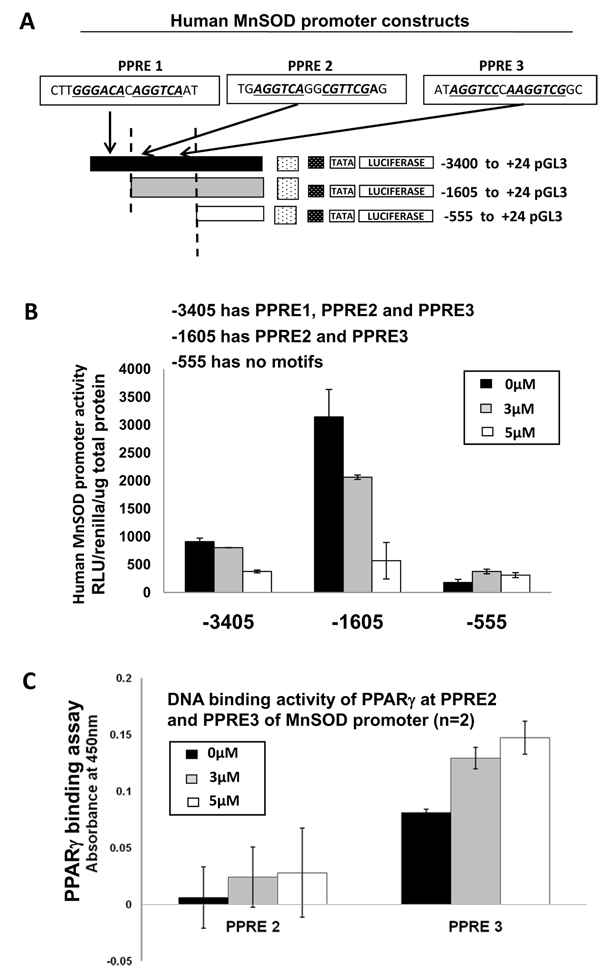
**PPARγ binds to PPRE3 in human MnSOD promoter**. (A) Schematic representations of full length human MnSOD promoter construct (-3400 to +24 pGL3) and two 5'-deletion constructs (-1605 to +24 pGL3; -555 to +24 pGL3) with location of PPRE1, PPRE2 and PPRE3 indicated. (B) Luciferase assay of MnSOD promoter activity in MDA-MB-231 transiently transfected with full length and 5'-deletion derivatives followed 5 μM and 10 μM 15 d-PGJ_2 _treatment for 16 h. *Renilla *plasmid was cotransfected to assess transfection efficiency. Results are expressed as RLU/renilla/μg total protein. Data represents the average +/-SD of two experiments done in duplicate. (C) No-shift DNA binding assay to assess DNA binding activity of PPARγ at PPRE2 and PPRE3 of MnSOD promoter as described in M&M. Results are expressed as absorbance readings at 450 nm. Data represents the average +/- SD of two experiments done in duplicate.

To determine which of the three PPREs within the human MnSOD is the true PPARγ binding site, we transfected MDA-MB-231 cells with a human MnSOD promoter construct (-3400 to +24, containing PPRE1, PPRE2, and PPRE3) and a 5' deletion construct (-1605 to +24) that lacks PPRE1 but has PPRE2 and PPRE3 (Figure 6A). Our results show both full length construct and the 5' deletion construct responded to 15 d-PGJ_2 _treatment in a dose-dependent decrease in promoter activity (Figure 6B). This seems to suggest that the bonafide PPRE would be either PPRE2 or PPRE3. Interestingly, a further 5' deletion construct (-555 to +24) that lacks all three PPREs did not decrease with 15 d-PGJ_2 _treatment, which suggests that 15 d-PGJ_2 _treatment on human MnSOD promoter activity is PPARγ-dependent (Figure 6B). To determine if PPRE2 or PPRE3 is the bonafide PPARγ binding site, using a NoShift assay with 5' biotinylated oligonucleotides for each of the two PPREs,(Figure 6C), we conclusively show that PPRE3 is indeed the PPARγ binding site within the human MnSOD promoter region, providing experimental proof for our prediction. Interestingly, PPRE3 also had the strongest predicted binding efficiency of 0.89 based on literature. More importantly, PPRE2 which was not predicted by PPRE (but was added as it was Alu element (Sx)) to bind PPARγ, our DNA binding assay indeed shows no significant binding by PPARγ to PPRE2 (Figure 6C).

## Discussion

Breast cancer is the third most common cancer globally and accounts for the highest morbidity and mortality. It is the second highest occurring cancer in women and one of the leading causes of death [26]. Although anti-estrogens have provided an effective endocrine therapy, a large number of patients acquiring resistance to these drugs pose a significant problem. Recently, a new approach using activators of peroxisome proliferator-activated receptor gamma (PPARγ) to inhibit proliferation and increase cell death in breast cancer cell lines has given hope to the development of a new class of anticancer drugs [27]. Interestingly, tumor breast tissue expresses PPARγ in amounts greater than normal breast epithelium, suggesting for a novel therapeutic approach for ligand-activated PPARγ as an anti-tumor agent in differentiation-based breast cancer therapy.

Normal functioning of cell metabolism occurs within a restricted intracellular pH (pH_i_) range. Regulation of pH_i _is accomplished via active extrusion of H^+ ^by the Na^+^/H^+ ^Exchangers (NHEs), a membrane antiporter expressed in a variety of cell types. The NHE family consists of ten isoforms, NHE1 to NHE10. Interestingly, apart from its role as a principal regulator of pH_i _and cell volume, the ubiquitously expressed NHE1 has been implicated in cell proliferation and transformation. Conversely, tumor cells deficient in NHE1 either fail to grow or show severely retarded growth when implanted in immuno-deficient mice [12]. More recently, we showed that down regulation of NHE1 expression by direct silencing of the gene expression or H_2_O_2 _treatment leads to cells' growth arrest and sensitization to etoposide or staurosporine [16,17].

Development of breast cancer has also been correlated to oxidative stress, brought about by alterations to the delicate balance between reactive oxygen species (ROS) and oxidative defenses [28,29] ROS stress seems to render cancer cells more dependent on MnSOD to protect them by maintaining cellular ROS balance. The importance of MnSOD became clearer when genetic knockout studies in mice indicate that MnSOD, but not other SODs, is essential for cell survival. Several recent studies reported that forced suppression of MnSOD expression by siRNA leads to decrease in breast cancer cells invasive property [30]and to sensitization of ovarian cancer cells to anti-cancer drugs [31]. Although the mouse MnSOD gene has been shown to be a PPARγ target gene [32], a direct association between PPARγ and human MnSOD from tumor cells' perspective has not been shown.

Taken together, our results suggest that NHE1 and MnSOD have PPRE binding motifs of high affinity. This confirms that these genes can be downregulated by PPARγ in presence of a ligand. Since, downregulation of NHE1 inhibits proliferation and increases cell death in breast cancer cell lines, the presented data provides new direction for development of a new class of anticancer drugs. Concurrently, downregulaion of MnSOD expression is reported to decrease cancer cells invasive property [30] and sensitizes cancer cells to anti-cancer drugs. Thus, the presented data suggests that PPARγ can regulate MnSOD in human cell linesPPARγ has been found to regulate MnSOD transcription by binding to a PPRE on the mouse MnSOD promoter region. This report for the first time suggests PPARγ activation and its involvement in downregulating MnSOD gene expression in human.

## Competing interests

The authors declare that they have no competing interests.

## Authors' contributions

G.V., S.M.K., and A.P.K conceived the idea of developing a PPRE motifs database. G.V. collected all motifs, constructed the database and wrote CGI-script for searching motifs. S.M.K. supervised the construction of the PPRE motifs database. A.P.K, M.V.C, and S.P. designed and supervised all experimental data. A.P.K. and S.Y.L performed validation experiments on NHE1 and MnSOD. S.M.K., G.V., A.P.K., and M.V.C. wrote the manuscript.

## Note

Other papers from the meeting have been published as part of *BMC Bioinformatics *Volume 10 Supplement 15, 2009: Eighth International Conference on Bioinformatics (InCoB2009): Bioinformatics, available online at http://www.biomedcentral.com/1471-2105/10?issue=S15.

## Supplementary Material

Additional file 1 List of DR1 and DR2 motifs from literature. Click here for file
